# Engineered Microorganisms for the Production of Food Additives Approved by the European Union—A Systematic Analysis

**DOI:** 10.3389/fmicb.2018.01746

**Published:** 2018-08-03

**Authors:** Nicolai Kallscheuer

**Affiliations:** Institute of Bio- and Geosciences, IBG-1: Biotechnology, Forschungszentrum Jülich GmbH, Jülich, Germany

**Keywords:** food additives, E numbers, European Union, microbial production, metabolic engineering, plant natural products

## Abstract

In the 1950s, the idea of a single harmonized list of food additives for the European Union arose. Already in 1962, the E-classification system, a robust food safety system intended to protect consumers from possible food-related risks, was introduced. Initially, it was restricted to colorants, but at later stages also preservatives, antioxidants, emulsifiers, stabilizers, thickeners, gelling agents, sweeteners, and flavorings were included. Currently, the list of substances authorized by the European Food Safety Authority (EFSA) (referred to as “E numbers”) comprises 316 natural or artificial substances including small organic molecules, metals, salts, but also more complex compounds such as plant extracts and polymers. Low overall concentrations of such compounds in natural producers due to inherent regulation mechanisms or production processes based on non-regenerative carbon sources led to an increasing interest in establishing more reliable and sustainable production platforms. In this context, microorganisms have received significant attention as alternative sources providing access to these compounds. Scientific advancements in the fields of molecular biology and genetic engineering opened the door toward using engineered microorganisms for overproduction of metabolites of their carbon metabolism such as carboxylic acids and amino acids. In addition, entire pathways, e.g., of plant origin, were functionally introduced into microorganisms, which holds the promise to get access to an even broader range of accessible products. The aim of this review article is to give a systematic overview on current efforts during construction and application of microbial cell factories for the production of food additives listed in the EU “E numbers” catalog. The review is focused on metabolic engineering strategies of industrially relevant production hosts also discussing current bottlenecks in the underlying metabolic pathways and how they can be addressed in the future.

## Introduction

### History of the approval of food additives in the european union

In industrial food production, consistent quality of foodstuff, and protection against contamination by harmful microorganisms must be guaranteed. For this purpose, a broad range of natural or artificial food additives are used in today's food applications, which not only serve for increasing the shelf life of foodstuff, but also for modifying its color, taste, odor, or texture. For preventing an arbitrary use of potentially harmful substances, there was strong desire toward establishing obligatory guidelines for the use of food additives. The additives sector has played a very important role in the approximation of food law in the European Union (EU). The first regulation, on which the six member states of the European Economic Community (EEC) (Belgium, Germany, France, Italy, Luxembourg, and the Netherlands) were able to agree, was the “Directive on food colorants” in 1962 (Directive 62/2645/EEC), which marks the beginning of a comprehensive harmonization effort. With this directive the E-classification system, a robust food safety system intended to protect consumers from possible food-related risks, was introduced. This classification system lists food additives approved by the EU (or its predecessor organizations) in form of “E numbers” allowing an unambiguous identification of a single compound (which might have several designations, e.g., trivial names), a set of chemically similar compounds or a plant extract.

In 1965, the regulation was extended to preservative substances which may be used in foodstuff (Directive 65/66/EEC). In a similar manner, other classes of food additives were included, e.g., anti-oxidants, emulsifiers, stabilizers, thickeners, and gelling agents in 1978 (Directives 78/663/EEC and 78/664/EEC) and sweeteners in 1994 (Directive 94/35/EC). In parallel, a number of additional intermediate steps in the form of European directives, e.g., for monitoring of purity criteria and for maintenance of national bans on the use of certain additives, was adopted. In 2008, the Framework Regulation (EC) No. 1333/2008 on food additives, and thus directly applicable law, was finally adopted and revised in 2010 in the Directive (EU) No. 257/2010. The principles described therein include health safety, technological necessity and prevention of misleading of food additives. In its current form the list of food additives approved by the EU includes 316 compounds, which are classified according to their major application as colors (E100–E199), preservatives (E200–E299), antioxidants and acidity regulators (E300–E399), thickeners, stabilizers, and emulsifiers (E400–E499), anti-caking agents (E500–E599), flavor enhancers (E600–E699), antibiotics (E700–E799), glazing agents, gases, and sweeteners (E900–E999) and additional additives (E1000–1599). Not all E numbers have been assigned and some E numbers were removed from the list.

### Sources for getting access to food additives approved by the EU

The compounds listed in the E numbers catalog comprise small organic molecules, metals, inorganic salts, but also polymers and complex compounds derived from plants or animals (e.g., beeswax, E901). There are in principle three sources of the approved compounds: extraction from the natural producer (e.g., plant material), chemical production or production in (engineered) microorganisms. Not every source is suitable for the production of each of the above-mentioned type of compounds, e.g., metals, inorganic salts, and synthetic colors cannot be produced by microorganisms, whereas complex plant-derived substances (e.g., Gummi arabicum, E414) can often not be produced by chemical synthesis. For this reason, this review article focuses on the production of natural small organic molecules, which are accessible through metabolic engineering of microorganisms either because they represent natural intermediates of their carbon metabolism or because they can be produced after functional introduction of heterologous pathways (Table 1).

**Table 1:** Microbially accessible compounds listed in the E numbers catalog sorted by application.

**E number**	**Compound**	**Compound class**
**COLORS**		
E100	Curcumin	Polyphenol
E101	Riboflavin (vitamin B2)	Nucleotide-derived compound
E160a	Carotenes	Terpenoid
E160b	Bixin, norbixin	Terpenoid
E160c	Capsanthin, capsorubin	Terpenoid
E160d	Lycopene	Terpenoid
E161b	Lutein	Terpenoid
E161g	Canthaxanthin	Terpenoid
E162	Betanin	Polyphenol
E163	Anthocyanins	Polyphenol
**PRESERVATIVES**		
E210-E213	Benzoic acid and salts	One-ring aromatic
E214-E219	4-Hydroxybenzoic acid esters	One-ring aromatic
E234	Nisin	Oligopeptide
E235	Natamycin	Macrolide
E270	Lactic acid	Carboxylic acid
E296	Malic acid	Dicarboxylic acid
E297	Fumaric acid	Dicarboxylic acid
**ANTI-OXIDANTS**		
E300-E302	Ascorbic acid and salts	Sugar acid
E307	α-Tocopherol	Terpenoid/phenol
E308	β-Tocopherol	Terpenoid/phenol
E308	δ-Tocopherol	Terpenoid/phenol
E310-E312	Gallic acid esters	One-ring aromatic
E330-E333	Citric acid and salts	Dicarboxylic acid
E334-E337, E354	Tartaric acid and salts	Dicarboxylic acid
E355-E357	Adipic acid and salts	Dicarboxylic acid
E363	Succinic acid	dicarboxylic acid
**THICKENERS, STABILIZERS**		
E420	Sorbitol	Sugar alcohol
E421	Mannitol	Sugar alcohol
E957	Thaumatin	Protein
E959	Neohesperidin dihydrochalcone	Polyphenol
E967	Xylitol	Sugar alcohol
E968	Erythritol	Sugar alcohol
E422	Glycerol	Short-chain alcohol
**ANTI-CAKING AGENTS**		
E570	Fatty acids	Carboxylic acid
E574-E579	Gluconic acid and salts	Sugar acid
**FLAVOR ENHANCERS**		
E620-E625	l-Glutamic acid and salts	Amino acid
E630-E633	Inosinic acid and salts	Nucleotide phosphate
E640	Glycine	Amino acid
**GLAZING AGENTS, SWEETENERS**		
E920	l-Cysteine	Amino acid
**ADDITIONAL ADDITIVES**		
E1105	Lysozyme	Protein
E1519	Benzyl alcohol	One-ring aromatic
E1520	1,2-Propanediol	Short-chain alcohol

In many cases, heterologous enzymes from plants were functionally introduced in microorganisms allowing for the production of natural compounds, which can otherwise only be obtained from plants (Marienhagen and Bott, 2013). A generalized strategy for extraction of natural products from plant material is in most cases not applicable as plants produce such compounds in low amounts and typically harbor complex mixtures of chemically very similar compounds. In addition, the concentrations are subject to regional and seasonal differences and several compounds are only produced under special environmental conditions (e.g., after infections). Although a few plant compounds can nowadays be obtained by direct extraction from plant material (Dai and Mumper, 2010), the above-mentioned drawbacks serve as a strong motivation for reconstructing plant pathways in a controllable and reliable metabolic environment in microbial cell factories (Milke et al., 2018).

The E numbers catalog also includes small organic molecules such as carboxylic acids and aromatic compounds, which are typically produced by the chemical industry. Here, in particular environmental concerns for the production from non-regenerative raw material and the use of toxic chemicals are forces toward establishing alternative production platforms. The fact that microorganisms are naturally capable of producing carboxylic acids and aromatic compounds was recognized as an excellent starting point for metabolic engineering work into this direction in the recent decades (Abbott et al., 2009; Gosset, 2009).

The E numbers catalog classifies food additives according to their major application in foodstuff. However, also distinct and metabolically rather unrelated classes of small organic molecules can have similar application ranges, e.g., aliphatic terpenoids and aromatic phenols are both used as anti-oxidants (Table 1). For this reason, compounds mentioned in the following sections are subdivided based on similar biosynthetic pathways or by use of the same precursor metabolites for production.

## Metabolic engineering for the microbial production of food additives

### General requirements for microorganisms used for production

**Figure 1:**
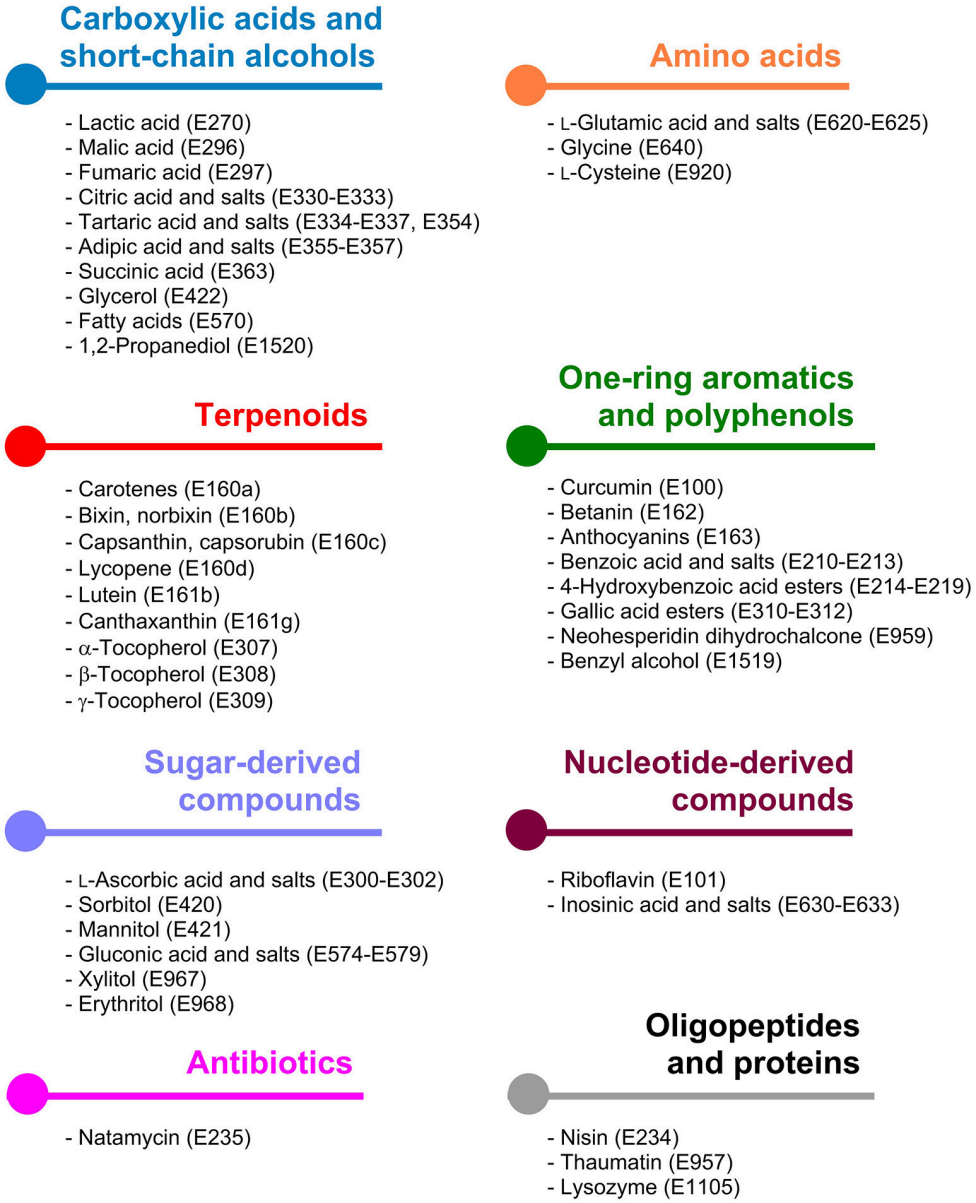
Classification of microbially accessible food additives according to the metabolic pathways being involved in their biosynthesis. Colors indicate the same compound class and are consistently used in Figures 2–**8**.

The underlying metabolic pathways relevant for the production of food additives have significant influence on the choice of the microorganism used for production and on the metabolic engineering strategies followed for estabilishing and improving production. For some products microorganisms already naturally overproducing the desired compound are tested. In contrast, for products which naturally do not occur in microorganisms it is reasonable to use microbial hosts, which can be easily genetically manipulated. General requirements for microbial production strains include fast growth, cultivation in cheap, and defined growth media to high biomass concentrations, non-pathogenicity, and in particular important when food additives are supposed to be produced the obtained product should have GRAS (“generally recognized as safe”) status. In this review article the categories are chosen based on related biosynthetic pathways as shown in Figure 1 and in each of the following sections the products are sorted by their respective E numbers.

### Carboxylic acids and short-chain alcohols

**Figure 2:**
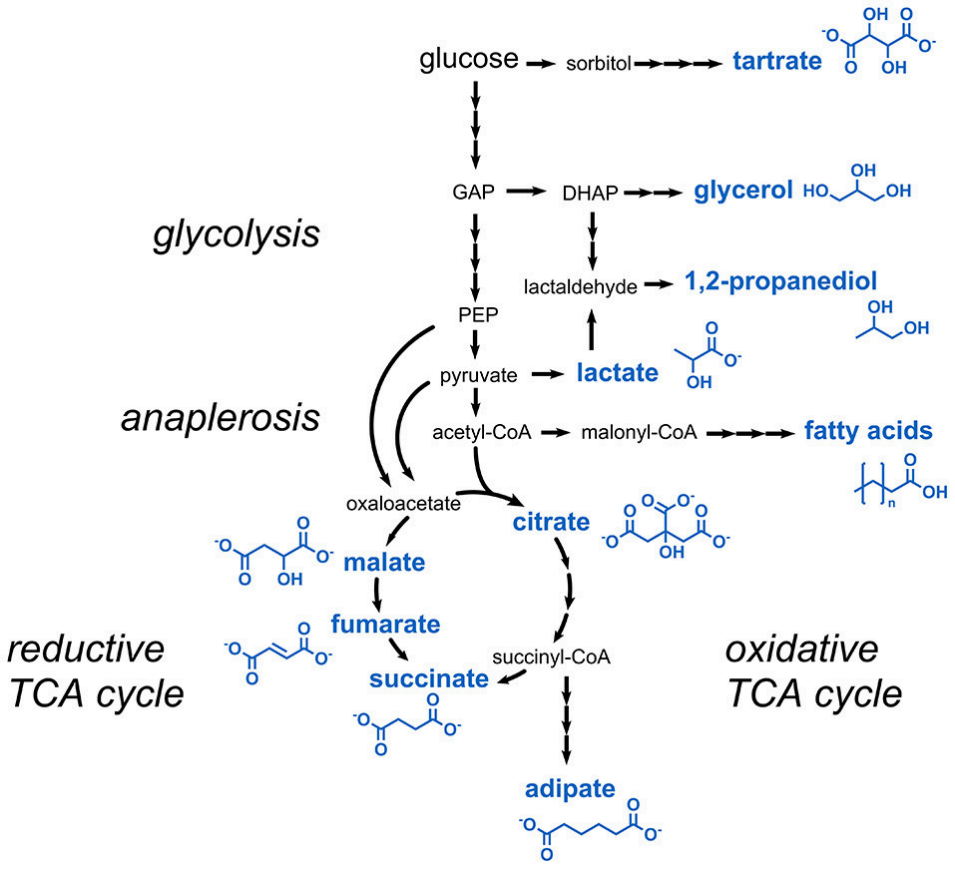
Metabolic pathways involved in the production of carboxylic acids and short-chain alcohols. Three arrows indicate several reaction steps which are not shown in detail. DHAP, dihydroxyacetone phosphate; GAP, glyceraldehyde 3-phosphate; PEP, phosphoenolpyruvate.

Aliphatic carboxylic acids are for the most part used as preservatives or acidity regulators. Most of the carboxylic acids listed in the E numbers catalog can be derived from glycolysis or the tricarboxylic acid (TCA) cycle (Figure 2). These comprise lactic acid, citric acid, succinic acid, fumaric acid, and malic acid.

Production of lactic acid (E270) was established in several microorganisms, e.g., lactic acid bacteria (*Lactobacillus* sp. and *Lactococcus* sp.), *Bacillus subtilis, Escherichia coli, Corynebacterium glutamicum*, yeasts, and microalgae (Abdel-Rahman et al., 2013). In addition to glucose also lignocellulosic biomass, agro-industrial, and food waste or glycerol were used as raw materials. Typically, yields of 0.85–0.98 g lactic acid per g carbon source were obtained. A maximal titer of 225 g/L lactic acid with a yield of 0.99 g/g was e.g., obtained from glucose during fed-batch cultivation of a non-engineered alkaliphilic *Bacillus* species (Meng et al., 2012). In addition to the use as food additive lactic acid is also used in large amounts for the production of the biodegradable polyester polylactic acid (Auras et al., 2011).

The TCA cycle intermediate malic acid (E296) is typically produced in *Aspergillus* species, e.g., *A. flavus, A. niger*, and *A. oryzae* (Battat et al., 1991; West, 2011). The latter was further engineered for increased production of malic acid from glycolysis-derived pyruvate by the reductive TCA cycle (Figure 2). An engineered strain overexpressing the genes coding for pyruvate carboxylase, malate dehydrogenase, and a C_4_-dicarboxylate transporter was capable of producing 154 g/L malic acid with a yield of 1.02 g/g glucose corresponding to 69% of the maximal theoretical yield (Brown et al., 2013). Similar metabolic engineering strategies were also followed in *S. cerevisiae*, which produced 59 g/L malic acid with a yield of 0.31 g malic acid per g glucose (Zelle et al., 2008). Malic acid production from glycerol was achieved in the plant pathogen *Ustilago trichophora* by combining adaptive laboratory evolution and metabolic engineering approaches (Zambanini et al., 2017). The highest malic acid titer obtained with the optimized strain was 134 g/L. In *E. coli*, the introduction of a mutation into the fumarate reductase gene along with the deletion of genes coding for two malic enzymes and lactate dehydrogenase led to the production of 34 g/L malic acid with a product yield of 1.06 g/g glucose (Zhang et al., 2011b). In very recent studies, additional strategies toward malic acid production in *E. coli* were tested and production from alternative carbon sources such as xylose was demonstrated (Li et al., 2018; Martinez et al., 2018).

For the production of fumaric acid (E297), which is directly formed from malic acid by fumarase activity, strains of *Rhizopus nigricans* or *R. oryzae* are applied as these fungi naturally overproduce fumaric acid. A titer of 85 g/L and yield of 0.85 g fumaric acid per g glucose were obtained during cultivation of *R. oryzae* self-immobilized on plastic discs of a rotary biofilm contactor (Cao et al., 1996). A succinate-producing *E. coli* mutant was turned into a fumarate producer by deletion of three fumarase-encoding genes (Li et al., 2014b). Additional strain engineering toward reducing the production of acetate led to an optimized strain producing 42 g/L fumaric acid from glycerol with 70% of the maximum theoretical yield.

Production of citric acid (E330) at industrial scale is typically conducted based on wild-type *A. niger* (Papagianni, 2007). This yeast naturally overproduces citric acid while the molecular mechanism for its accumulation and secretion is still not entirely understood (Karaffa and Kubicek, 2003). In a very recent study, an industrially used *A. niger* strain obtained from random mutagenesis, which is capable to produce 160 g/L citric acid, was analyzed by transcriptome analysis in comparison to strains producing lower amounts of citric acid (Yin et al., 2017). Significantly regulated genes coding for enzymes of glycolysis and the TCA cycle as well as for putative citrate transporters are the basis for a detailed analysis of the mechanism for citric acid accumulation and thus for future engineering work. *Yarrowia lipolytica* was recognized as an alternative microorganism for citric acid production from inexpensive carbon sources. Under nitrogen-limiting conditions production of 82 g/L citric acid was shown from glycerol-containing biodiesel waste (Kamzolova et al., 2015).

The dihydroxylated C_4_ dicarboxylic acid tartaric acid (E334) is structurally closely related to the TCA cycle intermediates succinic acid and malic acid, and can be converted to oxaloacetic acid in one single dehydration reaction (Hurlbert and Jakoby, 1965). Astonishingly, tartaric acid is not derived from a precursor of the TCA cycle but from a completely unrelated metabolic pathway. In higher plants, it was shown that tartaric acid is produced from l-ascorbic acid via 5-keto d-gluconic acid (DeBolt et al., 2006) (Figure 2). The latter is a natural product obtained from sugar oxidation in the acetic acid bacterium *Gluconobacter oxydans* subsp. *suboxydans*. It was shown that tartaric acid was spontaneously formed from 5-keto d-gluconic acid in culture medium for *G. oxydans* containing vanadium salts (Klasen et al., 1992). A titer of 2.1 g/L tartaric acid from 20 g/L of sorbitol was obtained with this organism (Chandrashekar et al., 1999). However, for commercial applications, large amounts of tartaric acid are obtained today as a by-product from wine industry or by chemical conversion of maleic acid or fumaric acid (Church and Blumberg, 1951; Zhang et al., 2011a).

The C_6_ dicarboxylic acid adipic acid (E355) serves as a major building block for the synthesis of nylon polymers, but is also used in foodstuff as acidulant. Adipic acid can be microbially accessed by several pathways, e.g., by the fatty acid synthesis pathway, by degradation of aromatic compounds, or starting from the TCA cycle intermediates succinyl-CoA or 2-oxoglutarate (Polen et al., 2013; Kallscheuer et al., 2017a) (Figure 2). Current efforts for establishing a bio-based production of adipic acid in microorganisms are summarized in a number of recent review articles (Deng et al., 2016; Kallscheuer et al., 2017b; Kruyer and Peralta-Yahya, 2017). The highest published titer for adipic acid of 2.5 g/L was obtained in *E. coli* (Cheong et al., 2016). The constructed strain harbors a heterologous catabolic pathway for adipic acid ultimately leading to succinyl-CoA, which was exploited in the reverse direction for adipic acid biosynthesis. The same pathway is present in the thermophilic bacterium *Thermobifida fusca* in which the overexpression of a gene for an endogenous adipyl-CoA dehydrogenase enabled the production of 2.2 g/L adipic acid without further strain engineering (Deng and Mao, 2015).

The TCA cycle intermediate succinic acid (E363) serves as a flavor enhancer for desserts, dry soups, and drink powder due to its mildly acidic and at the same time slightly salty flavor (Glassner and Datta, 1992). Succinic acid is typically produced via the reductive TCA cycle (Figure 2) under microaerobic or anaerobic conditions for achieving sufficient NADH supply. Several organisms were engineered for its production, e.g., *Actinobacillus succinogenes, Basfia succiniciproducens, Mannheimia succiniciproducens, E. coli, C. glutamicum*, and *S. cerevisiae* (Yan et al., 2014; Choi et al., 2016; Meng et al., 2016; Salvachúa et al., 2016; Lange et al., 2017; Mao et al., 2018). The tested strategies during strain engineering included increasing the flux into the pentose phosphate pathway for increasing NADH supply, elimination of competing pathways leading to acetate or lactate formation and overexpression of genes coding for succinate exporter proteins. With these strategies titers in the range of 30–90 g/L and yields of 0.6–1.3 g product per g carbon source were obtained (Ahn et al., 2016).

Nowadays, the triol glycerol (E422) is recognized as a cheap carbon source rather than as a product. This is due to its availability in large quantities as a by-product of biodiesel production (Quispe et al., 2013). However, in the 1990s in particular yeast was extensively engineered for production of glycerol and a maximal titer of 130 g/L and a yield of 0.63 g/g glucose was obtained in the osmotolerant yeast *Candida glycerinogenes* (Wang et al., 2001). In the recent years, there are still several studies reporting on glycerol production, e.g., in the model cyanobacterium *Synechocystis* sp. PCC6803 (Savakis et al., 2015), and also production in yeast strains is still further optimized (Tilloy et al., 2014; Yu et al., 2014; Murashchenko et al., 2016; Semkiv et al., 2017).

The E number E570 lists fatty acids and refers in particular to the long-chain fatty acid stearic acid, which is used as anti-caking agent or as plasticizer in chewing gum. For the production of fatty acids typically oleaginous yeasts such as *Y. lipolytica, Trichosporon dermatis*, or *Rhodosporidium toruloides* are employed, which are capable of accumulating long-chain fatty acids with a typical length of 16 or 18 carbon atoms. During fed-batch fermentations lipid contents of 25 to 68% (w/w) with a different composition of long-chain fatty acids were obtained (Papanikolaou et al., 2002; Li et al., 2007; Huang et al., 2012; Qiao et al., 2015). The key enzyme for further improving fatty acid synthesis is the acetyl-CoA carboxylase, which catalyzes the ATP-dependent carboxylation of acetyl-CoA to malonyl-CoA. This enzyme catalyzes the first committed step in the fatty acid synthesis pathways and is strictly regulated by different regulation mechanisms for avoiding fatty acid overproduction (Brownsey et al., 2006; Wei et al., 2016).

The short-chain alcohol 1,2-propanediol (1,2-PD, E1520, also known as propylene glycol) is mainly used as a carrier in the production of flavors for foodstuffs. Several microorganisms were found to be capable of fermenting sugars to 1,2-PD and two metabolic routes for its biosynthesis were identified (Saxena et al., 2010). 1,2-PD can either be derived from the glycolysis intermediate dihydroxyacetone phosphate, which is first dephosphorylated to methylglyoxal and subsequently reduced to lactaldehyde and finally to 1,2-PD (Figure 2). Alternatively, in the second pathway lactic acid is reduced to lactaldehyde, which is then further reduced to 1,2-PD. Already in the 1980s, non-engineered *Clostridium thermosaccharolyticum* strains were found to produce 7.9 g/L 1,2-PD with a yield of 0.27 g/g glucose via methylglyoxal (Cameron and Cooney, 1986). Functional introduction of this pathway in an *E. coli* strain optimized for glycerol assimilation allowed for the production of 5.6 g/L 1,2-PD from glycerol (Clomburg and Gonzalez, 2011). The second pathway for 1,2-PD production by degradation of lactic acid was identified in *Lactobacillus buchneri* (Elferink et al., 2001). In *E. coli*, lactaldehyde was identified as a side product from cleavage of l-fucose (Cocks et al., 1974). Lactaldehyde-derived production of 84 mg/L 1,2-PD was reported in a mutant strain of *E. coli* constitutively expressing a gene coding for a propanediol dehydrogenase during growth on l-fucose (Cocks et al., 1974). It turned out that the strain was also capable of utilizing 1,2-PD as sole carbon and energy source (Sridhara et al., 1969). Thus, a functional reversal of the underlying catabolic pathway (e.g., by using anaerobic cultivation conditions) might be the basis for establishing 1,2-PD production *via* lactic acid in *E. coli*.

### Amino acids

Three proteinogenic amino acids are approved by the EU as flavor enhancers, namely l-glutamic acid (E620), glycine (E640), and l-cysteine (E920). l-Glutamic acid and in particular its monosodium salt (E621) are the major compounds responsible for the “*umami*” taste of foodstuff. l-Cysteine is also used as flour treatment agent to improve baking functionality. All three amino acids can in principle be isolated from hydrolyzed protein or can be produced by engineered microorganisms. In case of glycine, both strategies are not followed for industrial production as this amino acid can be more easily produced by chemical synthesis (Zeng et al., 2016). Especially the expensive step for purification of the l-form from a racemic mixture of chemically synthesized amino acids is not required for glycine as it lacks stereogenic centers.

**Figure 3:**
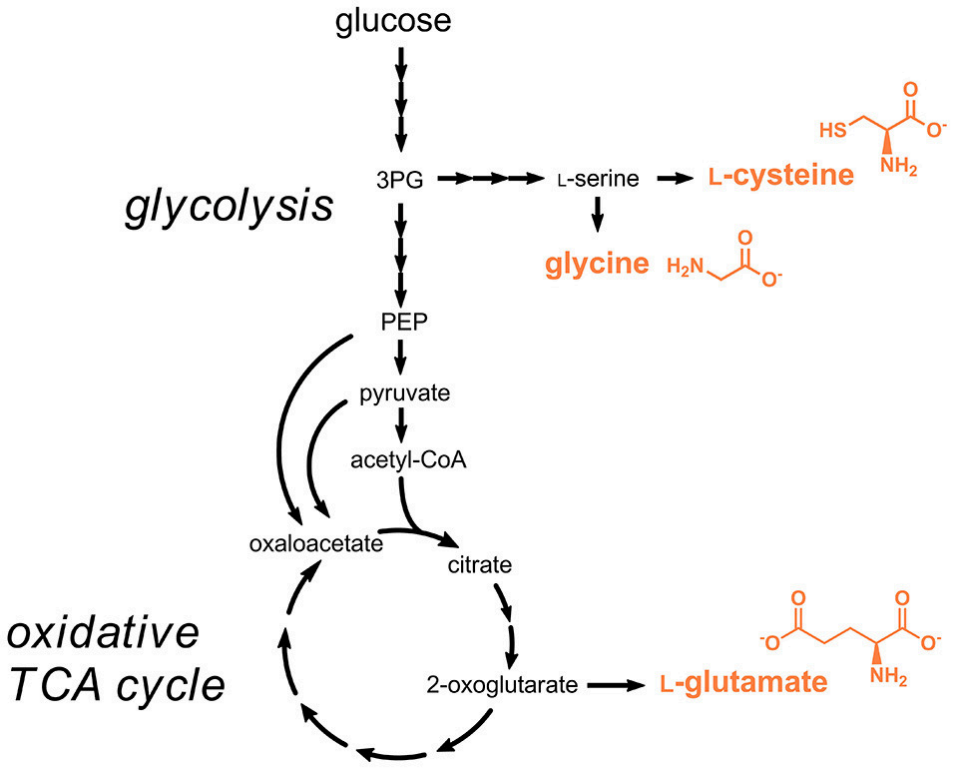
Production pathways for amino acids approved as food additives by the EU. Three arrows indicate several reaction steps which are not shown in detail. 3PG, 3-phosphoglycerate; PEP, phosphoenolpyruvate.

The l-glutamic acid-overproducing *C. glutamicum* was isolated in Japan in 1956 during a screening campaign for identifying glutamate-producing bacteria (Kinoshita et al., 1957). In the following decades the mechanism for glutamate secretion was investigated in detail and *C. glutamicum* was extensively engineering toward increased product titers (Hirasawa and Wachi, 2016). Combined activity during strain engineering and optimization of process conditions led to a strain capable of producing 100 g/L l-glutamic acid with a yield of 0.6 g per g glucose (Ault, 2004). Metabolic engineering efforts for increasing l-glutamic acid production in *C. glutamicum* are summarized in several books and review articles (Kimura, 2003; Eggeling and Bott, 2005; Sano, 2009). In recent years, alternative organisms such as *Pantoea ananatis* were also found to be “talented” producers of l-glutamic acid (Katashkina et al., 2009).

l-Cysteine is nowadays for the most part obtained from protein hydrolysis obtained from animal material, e.g., poultry feathers. The natural biosynthesis pathway starting from l-serine and acetyl-CoA was used for engineered l-cysteine production in *E. coli* and *C. glutamicum* (Wada and Takagi, 2006) (Figure 3). The rate-limiting reaction in the pathway is the initial step catalyzed the serine *O*-acetyltransferase (CysE), which is strictly feedback-inhibited by l-cysteine with enzyme inhibition constants in the micromolar range (Denk and Böck, 1987). Metabolic engineering efforts focused on introducing mutations in the respective gene *cysE* for obtaining less feedback-sensitive enzymes (Kai et al., 2006). By functional introduction of heterologous feedback-insensitive CysE isoenzymes from *Arabidopsis thaliana* into *E. coli* an l-cysteine titer of 1.7 g/L was obtained (Takagi et al., 1999). In a subsequent study, deletion of the gene *yciW*, which encodes an oxidoreductase putatively involved in l-cysteine metabolism in *E. coli*, led to an increased production of l-cysteine, however the titer of 1.7 g/L already obtained in 1999 was not exceeded (Kawano et al., 2015; Takagi and Ohtsu, 2016). Very recently, additional overexpression of the genes coding for SerA (3-phosphoglycerate dehydrogenase), SerB (phosphoserine phosphatase), and SerC (phosphoserine aminotransferase) involved in the synthesis of the precursor l-serine and deletion of genes coding for enzymes involved in the degradation of l-serine and l-cysteine was tested (Liu et al., 2018). The optimized strain produced 5.1 g/L l-cysteine during fed-batch fermentations.

### Terpenoids

**Figure 4:**
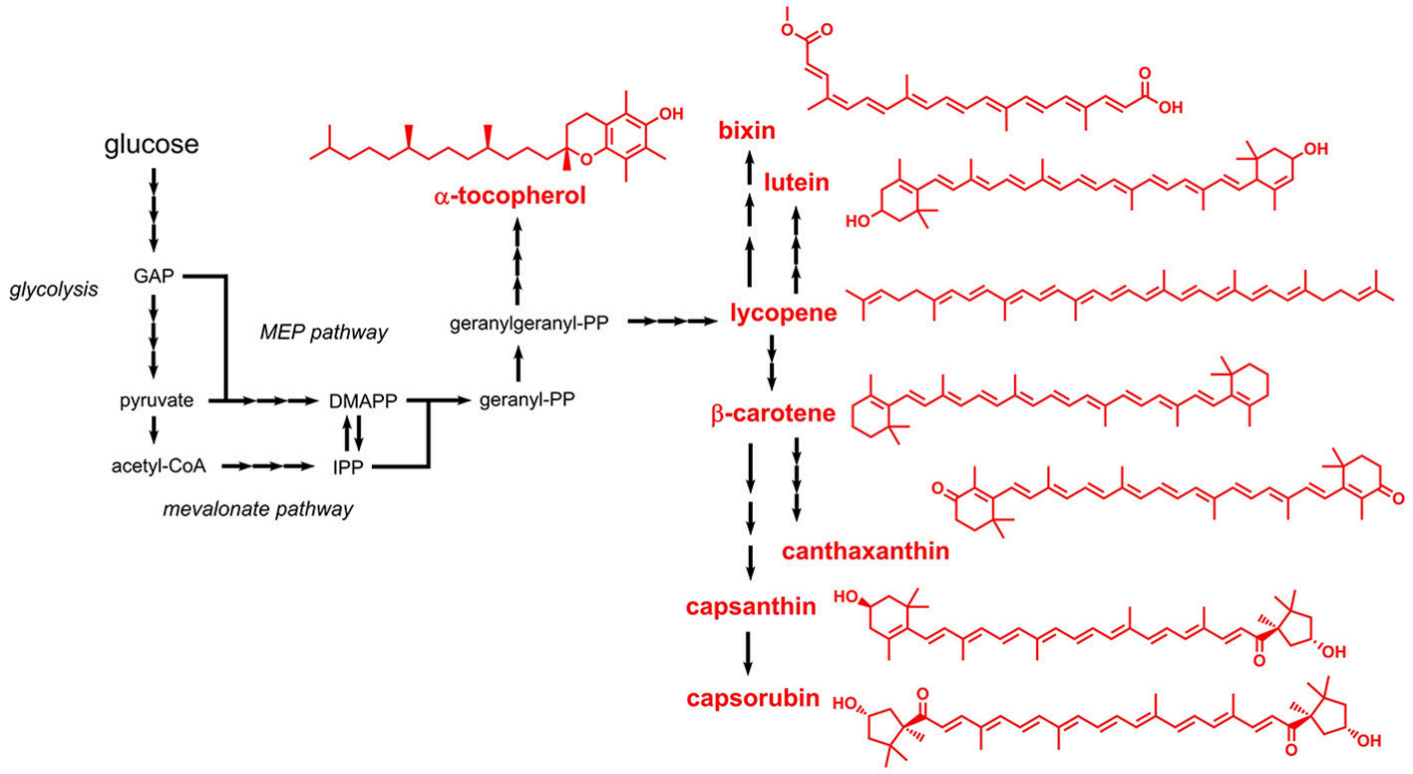
Biosynthetic pathways leading to terpenoids approved as food additives by the EU. Three arrows indicate several reaction steps which are not shown in detail. GAP, glyceraldehyde 3-phosphate; MEP, methyl erythritol phosphate; DMAPP, dimethylallylpyrophosphate; IPP, isopentenylpyrophosphate.

Terpenoids (also referred to as isoprenoids) are mostly aliphatic compounds derived from units of isoprene (2-methyl-1,3-butadiene). Most of the terpenoids listed in the E numbers catalog are used as colors and antioxidants. Based on the number of condensed isoprene units (isoprene: C_5_) the products are designated monoterpenes (C_10_), sesquiterpenes (C_15_), diterpenes (C_20_), or tetraterpenes (C_40_). The isoprene units are naturally supplied in form of the two isomers dimethylallyl pyrophosphate (DMAPP) or isopentenyl pyrophosphate (IPP). Two independent pathways responsible for DMAPP/IPP formation were identified: the mevalonate pathway and the methyl-d-erythritol 4-phosphate (MEP) pathway (non-mevalonate pathway) (Goldstein and Brown, 1990; Eisenreich et al., 2004) (Figure 4). The mevalonate pathway produces DMAPP/IPP from three molecules of acetyl-CoA, whereas the MEP pathway requires pyruvate and glyceraldehyde 3-phosphate. The linear, non-cyclic precursor compounds, from which all terpenoids with the corresponding length derive, are geranyl pyrophosphate (C_10_), farnesyl pyrophosphate (C_15_), geranylgeranyl pyrophosphate (C_20_), and phytoene (C_40_). These precursors are cyclized by downstream enzymes and are often further modified, e.g., by hydroxylation reactions.

Lycopene (E160d) is a central tetraterpene, which represents the precursor for biosynthesis of β-carotene (E160a), capsanthin and capsorubin (both E160c), lutein (E161b), and canthaxanthin (E161g) (Figure 4). Before starting metabolic engineering toward lycopene production in *E. coli*, the DMAPP/IPP-forming mevalonate pathway was reconstituted *in vitro* and the steady-state kinetic and biochemical parameters were analyzed (Zhu et al., 2015). Subsequent coupling of the optimized pathway with downstream enzymes required for converting DMAPP/IPP to lycopene led to an *E. coli* strain producing 1.4 g/L lycopene during fed-batch fermentation. Similar titers in the range of 0.9 - 1.1 g/L lycopene were also obtained in related studies performed with *E. coli* as production host (Zhang et al., 2015b; Xu et al., 2018). Alternative hosts for lycopene production include e.g., *Blakeslea trispora* and *Y. lipolytica* (Xu et al., 2007; Matthäus et al., 2014), which are also subject of a recent review article on engineered lycopene production (Hernández-Almanza et al., 2016).

Production of lycopene-derived β-carotene was shown in engineered yeasts such as *S. cerevisiae* and *Rhodotorula glutinis* (Bhosale and Gadre, 2001; Li et al., 2013). In *S. cerevisiae*, genes from the carotenoid-producing yeast *Xanthophyllomyces dendrorhous* were expressed, which led to the production of 5.9 mg β-carotene per g dry weight (Verwaal et al., 2007). One of the introduced genes codes for a 3-hydroxy-3-methylglutaryl-CoA reductase which catalyzes the rate-limiting step in the mevalonate pathway supplying the precursors DMAPP/IPP (Chappell et al., 1995). The entire mevalonate pathway and the β-carotene biosynthetic pathway were also functionally introduced into *E. coli* (Yoon et al., 2009). In this study, the best-performing strain with plasmid-borne expression of 10 heterologous genes produced 0.47 g/L β-carotene in complex medium with 2% glycerol.

Apocarotenoids such as bixin and norbixin (E160b) are obtained by oxidative cleavage of lycopene (Figure 4). Both compounds were produced by functional introduction of the lycopene cleavage dioxygenase from the plant achiote (*Bixa orellana*) together with an aldehyde dehydrogenase and a carboxyl methyltransferase into an engineered lycopene-producing *E. coli* strain (Bouvier et al., 2003). A production level of 5 mg bixin/g dry weight was obtained.

Capsanthin and capsorubin (E160c) belong to the class of xanthophylls (oxygen-containing carotenoids) and are the major carotenoids in red pepper fruits (Lefebvre et al., 1998). Both compounds can be produced from lycopene by six reactions steps including two cyclization and two hydroxylation steps and subsequent epoxidation and de-epoxidation. Production of capsanthin and capsorubin has not been achieved in microorganisms until today, but strategies for production in *E. coli* were proposed (Misawa, 2013).

Lutein (E161b), which is also a plant-derived xanthophyll, is produced from lycopene by two cyclization and two hydroxylation reactions (Kim and DellaPenna, 2006). Biotechnological lutein production focused exclusively on the use of microalgae, which naturally produce lutein (Fernández-Sevilla et al., 2010). It turned out that lutein production capabilities strongly depend on environmental and operating factors. A third xanthophyll, canthaxanthin (E161g), is naturally produced from β-carotene by two reaction steps, which are both catalyzed by the β-carotene ketolase CrtW. Several microorganisms naturally producing canthaxanthin were identified, e.g., *Haloferax alexandrinus, Gordonia jacobaea*, and *Dietzia natronolimnaea* (de Miguel et al., 2000; Asker and Ohta, 2002; Gharibzahedi et al., 2012). With mutated strains and by choosing cultivation conditions promoting canthaxanthin production, product titers in the range of 2–8 mg/L were obtained (Gharibzahedi et al., 2012; Rostami et al., 2014). The β-carotene ketolase gene *crtW* from *Agrobacterium aurantiacum* was already functionally expressed in an *E. coli* strain engineered for β-carotene production, which led to the production of detectable amounts of canthaxanthin in this organism (Misawa et al., 1995).

Some carotenoid biosynthetic pathways lead to the production of aromatic compounds, e.g., okenone and isorenieratene (these compounds are not listed in the E numbers catalog). In such cases the aliphatic rings, e.g., in β-carotene, are oxidized to aromatic (benzene) rings. Tocopherols (E307, E308, and E309) also contain an aromatic ring but represent an exception to the above-mentioned biosynthesis strategy. Tocopherols can be classified as terpenoids because they are derived from geranylgeranyl pyrophosphate (Figure 4). However, the aromatic ring present in tocopherol is not formed during terpenoid biosynthesis but is obtained from homogentisate (2,5-dihydroxyphenylacetate), which in turn is produced from the aromatic amino acid l-tyrosine (Arias-Barrau et al., 2004). Thus, microbial production of tocopherol requires engineering of two unrelated pathways, namely the terpenoid biosynthetic pathway and the l-tyrosine-forming shikimate pathway. This might be a reason why tocopherol production focused on natural producers, namely photosynthetic microorganisms such as *Dunaliella tertiolecta* and *Euglena gracilis* (Tani and Tsumura, 1989; Carballo-Cárdenas et al., 2003). In *E. gracilis*, a titer of 144 mg/L α-tocopherol was achieved.

### One-ring aromatics and polyphenols

**Figure 5:**
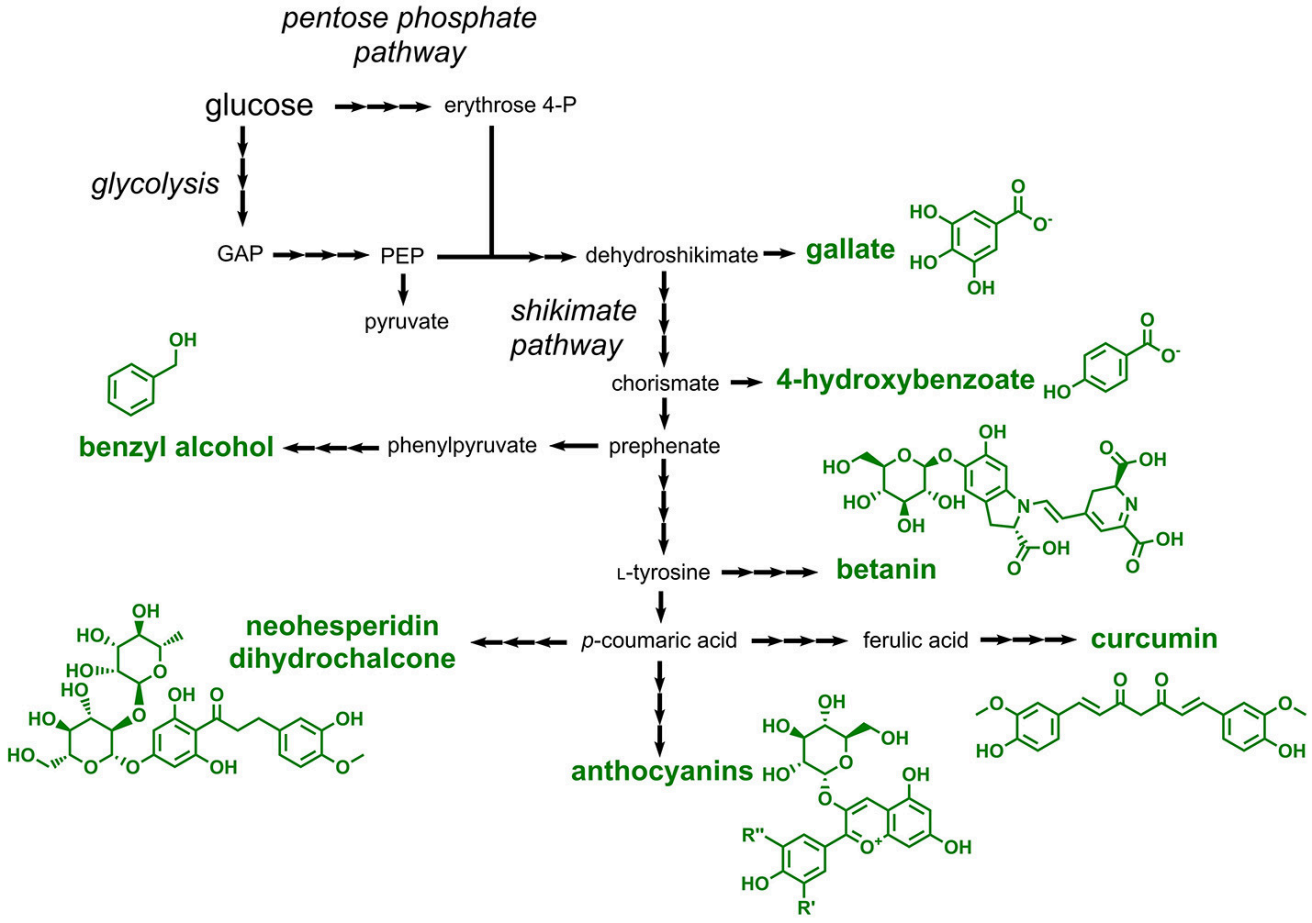
Pathways involved in the biosynthesis or aromatic food additives approved by the EU. Three arrows indicate several reaction steps which are not shown in detail. GAP, glyceraldehyde 3-phosphate; PEP, phosphoenolpyruvate.

Aromatic compounds approved as food additives by the EU can be classified into two large groups: one-ring aromatics and polyphenols. One-ring aromatics are typically benzoic acid derivatives, while polyphenols comprise a large group of structurally diverse and more complex secondary metabolites in plants (Quideau et al., 2011). All compounds of these two classes are derived from the aromatic amino acid-forming shikimate pathway, which requires the pentose phosphate pathway-derived erythrose 4-phosphate and glycolysis-derived phosphoenolpyruvate as precursors (Figure 5). One-ring aromatics are typically produced from intermediates of the shikimate pathway (e.g., shikimate or chorismate), whereas all polyphenols are derived from phenylpropanoids, which in turn are produced by deamination of the aromatic amino acids l-phenylalanine or l-tyrosine (Vogt, 2010) (Figure 5).

Four different one-ring aromatics, which can be accessed with microorganisms, are listed as E numbers: benzoic acid and different salts derived thereof (E210–E213), 4-hydroxybenzoic acid esters (E214–E219), gallic acid esters (E310–E312), and benzyl alcohol (E1519). Although a natural pathway for the production of benzoic acid by chain-shortening of the phenylpropanoid cinnamic acid was identified in plants (Moerkercke et al., 2009), this compound was not produced in engineered microorganisms until today. This is probably due to the fact that benzoic acid can be easily obtained from petroleum-derived toluene (Kaeding et al., 1965). In contrast, several microorganisms were engineered for the production of 4-hydroxybenzoic acid and gallic acid (3,4,5-trihydroxybenzoic acid), which are direct precursors for the corresponding methyl-, ethyl- and propyl esters approved as food additives. 4-hydroxybenzoic acid is microbially produced by cleavage of the shikimate pathway intermediate chorismate (Siebert et al., 1994) (Figure 5). In addition to the functional introduction of the required chorismate pyruvate lyase the feedback-regulation at the initial step of the shikimate pathway needs to be abolished. For this purpose, feedback-resistant enzymes catalyzing this initial step are applied (Weaver and Herrmann, 1990; Fukuda et al., 1991; Jossek et al., 2001). By following this strategy, 4-hydroxybenzoic acid was produced e.g., in *E. coli, C. glutamicum, S. cerevisiae*, and *Pseudomonas putida* (Meijnen et al., 2011; Noda et al., 2016; Averesch et al., 2017; Kallscheuer and Marienhagen, 2018). Very recently, a titer of 36.6 g/L and a yield of 0.31 g/g glucose was obtained in engineered *C. glutamicum* during fed-batch fermentation (Kitade et al., 2018). An *E. coli* strain overproducing the shikimate pathway intermediate 3-dehydroshikimate was capable of producing 20 g/L gallic acid, when an endogenous gene coding for a dehydroshikimate dehydrogenase was overexpressed (Kambourakis et al., 2000). 114 mg/L benzyl alcohol was produced from glucose in *E. coli* using a non-natural pathway starting from the shikimate pathway intermediate phenylpyruvate (Pugh et al., 2015).

Plant-derived polyphenols present in fruits and vegetables are part of our daily diet. Thus, it is surprising that only four polyphenols are approved as food additives by the EU. These include curcumin (E100, a yellow colorant), betanin (E162, a red colorant), anthocyanins (E163, a large class of plant colorants), and neohesperidin dihydrochalcone (E959, a natural sweetener).

Curcumin is produced starting from the phenylpropanoid *p*-coumaric acid, which in turn is obtained from the non-oxidative deamination of l-tyrosine (Katsuyama et al., 2009). *p*-Coumaric acid is first converted to the phenylpropanoid ferulic acid by hydroxylation and subsequent *O*-methylation. Ferulic acid is then CoA-activated by the activity of a CoA-ligase. The key enzyme curcumin synthase subsequently condenses the CoA-thioesters of ferulic acid and of a diketide derived from ferulic acid yielding curcumin. Curcumin production in the range of 60 mg/L from supplemented ferulic acid was demonstrated in *E. coli* by expression of heterologous genes coding for the CoA ligase and the curcumin synthase (Katsuyama et al., 2008). In a more recent study, curcumin production with a titer of 0.6 mg/L was achieved in *E. coli* starting from l-tyrosine (Wang et al., 2015).

Betanin (E162) is a glycosylated betacyanine, which is a major compound present in beetroot. This natural red dye is produced starting from l-tyrosine, which is first hydroxylated yielding l-3,4-dihydroxyphenylalanine (also known as Levodopa or l-DOPA). By the activity of two independent enzymes one molecule of l-DOPA is converted to cyclo-DOPA (precursor 1) and a second one to betalamic acid (precursor 2). Precursor 1 and 2 are subsequently ligated yielding betanidin, which is subsequently 5-*O*-glycosylated to betanin (Tanaka et al., 2008). The entire pathway was recently reconstructed in *S. cerevisiae* by functional expression of genes from various plants (Grewal et al., 2018). The constructed strains were not only capable of producing 17 mg/L betanin, but also converted alternative aromatic amines (instead of cyclo-DOPA) to the corresponding betacyanine dyes.

In addition to betanin, also plant-derived anthocyanins (E163) find an application as colorants in foodstuff. For the synthesis of anthocyanins the l-tyrosine-derived and CoA-activated phenylpropanoid *p*-coumaroyl-CoA acid is first condensed with three molecules of malonyl-CoA yielding a tetraketide intermediate, which is cyclized to the compound naringenin chalcone. All steps during this reaction, the malonyl-CoA-dependent chain elongation as well as the cyclization step, are catalyzed by chalcone synthases (Ferrer et al., 1999). By the activity of chalcone isomerases, naringenin chalcone is subsequently isomerized to the flavanone naringenin. Naringenin, the first compound in the pathway harboring the typical flavonoid core structure, is then further converted by plant dioxygenases and reductases giving rise to the anthocyanidin pelargonidin, which is further stabilized by *O*-glycosylation at C3 (the glycosylated anthocyanidin is then designated anthocyanin). Taken together, the overall pathway from *p*-coumaric acid includes seven reaction steps (1: CoA activation, 2: chain elongation and cyclization, 3: isomerization, 4: hydroxylation, 5: reduction, 6: oxidation, 7: glycosylation) (Figure 5). The resulting pelargonidin-3-*O*-glucoside can be further converted to related anthocyanins by ring hydroxylation or *O*-methylation reactions. For achieving microbial anthocyanin production in particular *E. coli* strains were constructed (Yan et al., 2005, 2008; Lim et al., 2015). Recently, the production of 9.5 mg/L pelargonidin-3-*O*-glucoside (callistephin) from glucose was achieved by a co-culture of four different *E. coli* strains together expressing 15 heterologous genes (Jones et al., 2017).

Neohesperidin dihydrochalcone (E959) is a rutinosylated chalcone, i.e., it is decorated with rutinose (rhamnose-α-1,6-glucose) (Figure 5). This chalcone is present in citrus fruits and is applied as a natural sweetener in beverages, yogurt and ice cream. Neohesperidin dihydrochalcone was not produced in engineered microbes so far, but a very similar compound, naringin dihydrochalcone, which differs from neohesperidin dihydrochalcone only by one *O*-methyl-group, was obtained in engineered *S. cerevisiae* (Eichenberger et al., 2017). The constructed strain heterologously expressed nine genes and produced 12 mg/L naringin dihydrochalcone from glucose. Based on these results a microbial production of neohesperidin dihydrochalcone can be achieved by including an additional *O*-methyltransferase capable of converting naringin dihydrochalcone to neohesperidin dihydrochalcone.

### Sugar-derived compounds

**Figure 6:**
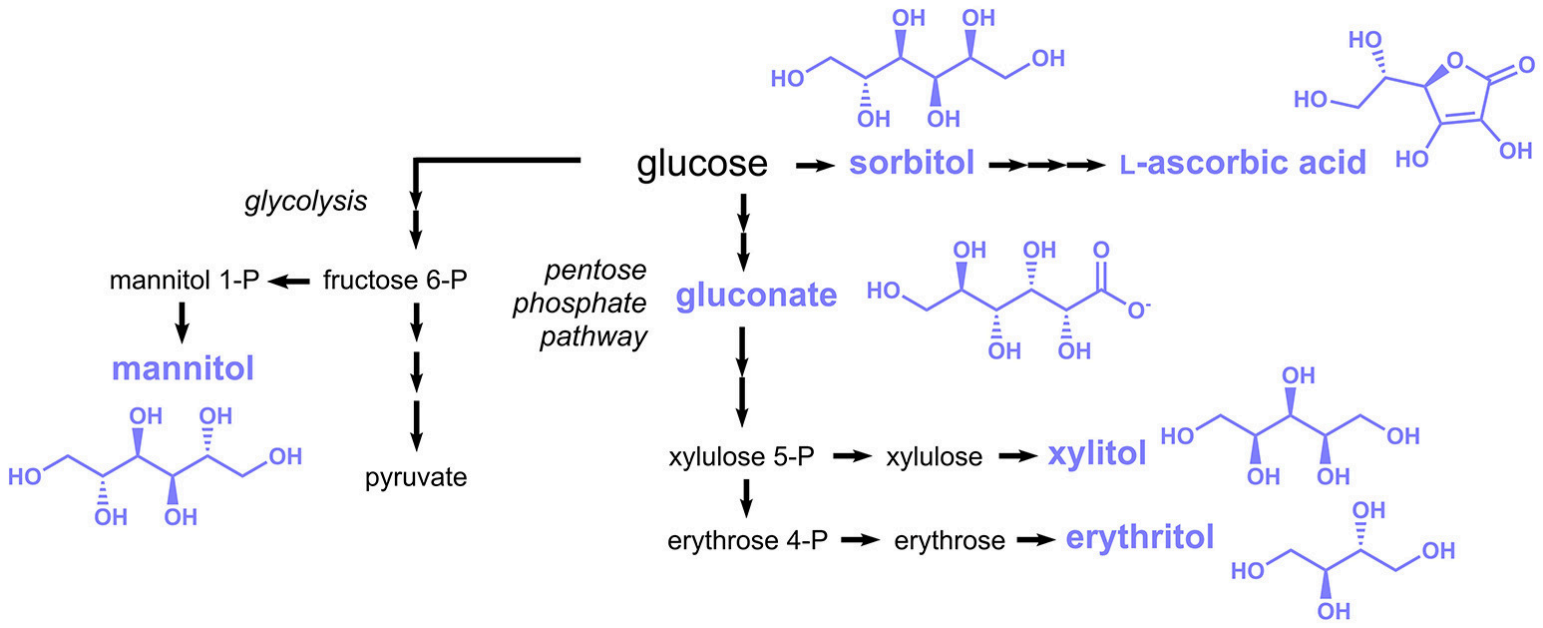
Biosynthesis of sugar-derived compounds approved as food additives by the EU. Three arrows indicate several reaction steps which are not shown in detail.

Subject of this section are food additives listed in the E numbers catalog, which are more or less directly derived from the sugar metabolism. Many different compounds can in principle be “derived from sugar”; hence only compounds derived from glycolysis and the pentose phosphate pathway are included here (Figure 6).

l-Ascorbic acid (E300, also known as vitamin C) is a vitamin traditionally consumed with fruits and vegetables, which is today produced at the scale of more than 100,000 tons per year (Pappenberger and Hohmann, 2013). l-Ascorbic acid is also extensively added to foods, beverages and pharmaceuticals. The established industrial production process of l-ascorbic acid is based on d-sorbitol or sorbose, which are first oxidized to 2-keto-l-gulonic acid by *Gluconobacter oxydans* or *Ketogulonicigenium vulgare*, repectively and then chemically converted to l-ascorbic acid (Pappenberger and Hohmann, 2013). *G. oxydans* was capable to produce 130 g/L 2-keto-l-gulonic acid from 150 g/L sorbitol (Saito et al., 1997), while a titer of 60 g/L was achieved from 70 g/L sorbose in *K. vulgare* (Ning et al., 1988).

Sorbitol (E420), the precursor for industrial l-ascorbic acid production, is also a food additive approved by the EU and is mainly used as natural sweetener, e.g., in chewing gum. Although sorbitol is traditionally synthesized by catalytic hydrogenation of glucose (Kusserow et al., 2003), it was also produced in engineered microorganisms from glucose in a single reduction step. In *Zymomonas mobilis* the enzyme glucose-fructose oxidoreductase was shown to catalyze the reduction of glucose to sorbitol by simultaneously oxidizing fructose to gluconolactone (Zachariou and Scopes, 1986). Not surprisingly, this organism was exploited for the production of sorbitol, in particular in the 1990s (Silveira and Jonas, 2002). In more recent studies also alternative hosts were applied, e.g., the cyanobacterium *Synechocystis* sp. and the lactic acid bacterium *Lactobacillus plantarum* (Jan et al., 2017; Chin et al., 2018).

Additional polyols with applications similar to sorbitol comprise mannitol (E421), xylitol (E967), and erythritol (E968) (Figure 6). These are also produced industrially by catalytic hydrogenation (Schiweck et al., 2000), but several microorganisms were engineered toward their production. Mannitol is typically produced in lactic acid bacteria, e.g., *Lactococcus lactis* or *Lactobacillus reuteri* via the glycolysis intermediate fructose-6-phosphate, which is reduced to mannitol-1-phosphate and subsequently dephosphorylated. Yields of 0.5–0.6 g mannitol per g glucose or fructose were obtained (Song and Vieille, 2009). A constructed *C. glutamicum* strain expressing genes coding for a mannitol dehydrogenase from *Leuconostoc pseudomesenteroides* and a glucose/fructose transporter from *Z. mobilis* was capable to produce 87 g/L mannitol from 94 g/L fructose (Bäumchen and Bringer-Meyer, 2007). The xylose reductase of *Pichia stipitis* was introduced into *S. cerevisiae*, which led to a xylose conversion rate of 95% in the recombinant strain (Hallborn et al., 1991). In a more recent study, the sugar import of a *Kluyveromyces marxianus* strain harboring a xylose reductase from *Neurospora crassa* was engineered and a final titer of 312 g/L xylitol was obtained during fed-batch fermentation (Zhang et al., 2015a).

For the production of erythritol, erythrose 4-phosphate (E4P) derived from the pentose phosphate pathway is first dephosphorylated to erythrose and subsequently reduced to erythritol (Figure 6). In some microorganisms, e.g., lactic acid bacteria, E4P is first reduced to erythritol 4-phosphate and then dephosphorylated. Yields of 0.3 to 0.4 g/g glucose and product titers in the range of 120–250 g/L were obtained in microorganisms engineered toward erythritol production (Moon et al., 2010).

Oxidation of glucose at the C1-atom leads to gluconic acid (E574) (Figure 6), which is used in food applications as an acidity regulator. The enzymatically-driven oxidation reaction can either be coupled to the reduction of a cofactor, e.g., NAD(P)^+^, FAD or a quinone (glucose 1-dehydrogenase) or alternatively molecular oxygen can serve as oxidant (glucose oxidase). The latter reaction also yields H_2_O_2_ as byproduct, which needs to be rapidly detoxificated by a catalase. Production of gluconic acid was achieved in an *A. niger* strain with high activity of glucose oxidase and catalase (Znad et al., 2004). After 60 h of cultivation a nearly complete conversion of 150 g/L glucose to 150 g/L gluconic acid was observed. Further optimization of the process conditions reduced the production time to 15 h and improved the yield and final titer to 1.05 g/g and 311 g/L respectively (Lu et al., 2015).

### Nucleotide-derived compounds

**Figure 7:**
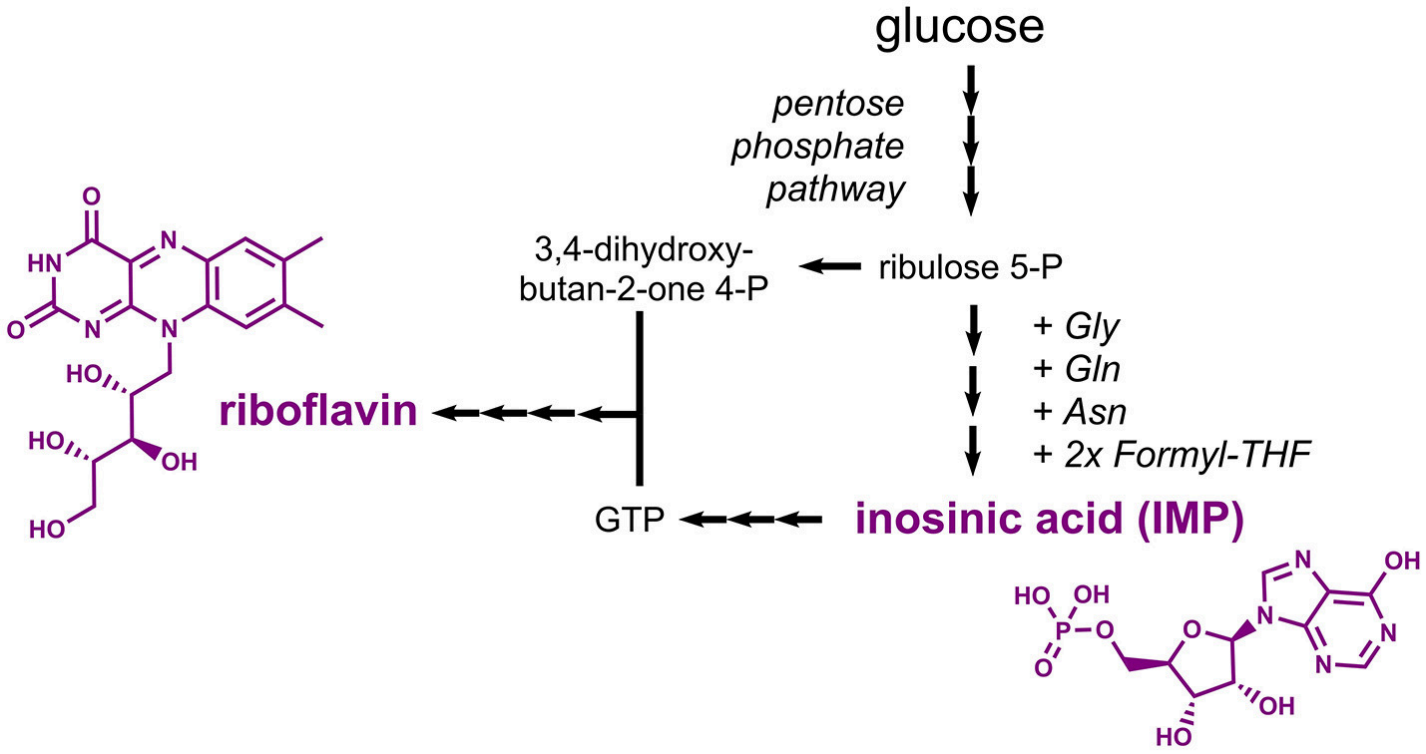
Pathways leading to production of the nucleotide-derived compounds inosinic acid and riboflavin. Three arrows indicate several reaction steps which are not shown in detail. Gly, glycine; Gln, glutamine; Asn, asparagine; THF, tetrahydrofolate; IMP, inosine monophosphate; GTP, guanosine triphosphate.

Riboflavin (E101, vitamin B_2_), a yellow colorant, and inosinic acid (E630), a flavor enhancer, are compounds derived from the nucleotide metabolism. Biosynthesis of riboflavin requires two precursors, namely the pentose phosphate pathway intermediate ribulose 5-phosphate and the nucleotide guanosine 5'-triphosphate (GTP), which is derived from the purine metabolism (Figure 7). Inosinic acid, which is also designated inosine 5'-monophosphate (IMP), is a pathway intermediate in the purine metabolism leading to biosynthesis of GTP. Thus, it is not surprising that similar metabolic engineering strategies were followed for establishing production of riboflavin and inosinic acid.

Microbially produced riboflavin is typically obtained from the filamentous fungus *Ashbya gossypii* or from the yeast *Candida famata* as both are natural riboflavin overproducers (Stahmann et al., 2000). *A. gossypii* was extensively engineered toward increased riboflavin titers in the recent years. To this end, rational approaches were followed to increase the carbon fluxes into the purine and riboflavin biosynthetic pathways. It turned out that reduced expression of the adenylosuccinate synthase gene *ADE12* led to an increased flux from IMP into the guanosine monophosphate (GMP) branch and reduced the competing flux into the adenosine monophosphate (AMP) branch. The overexpression of five genes coding for enzymes of the riboflavin synthesis pathway in a strain with reduced *ADE12* expression allowed for the production of 0.52 g/L riboflavin (Ledesma-Amaro et al., 2015). Very recently, the riboflavin production capabilities were further improved based on a ^13^C metabolic network analysis, which improved the final titer by 45% (the actual titer is not mentioned) (Schwechheimer et al., 2018). Metabolic engineering work in *E. coli* focused on overexpression of the native riboflavin biosynthesis genes *ribABDEC* along with additional modifications leading to reduced by-product formation and an increased flux into the pentose phosphate pathway (Lin et al., 2014). The best strain produced 2.7 g/L riboflavin with a yield of 0.14 g/g glucose. In engineered *B. subtilis* strains, riboflavin titers of 4.9 g/L were obtained (Wang et al., 2014).

Already in the 1960s, it was found that an adenine-auxotrophic *Micrococcus glutamicus* strain accumulated inosinic acid to a concentration of 0.75 g/L (IMP Na_2_ x 7.5 H_2_O) (Nakayama et al., 1964). Production was also achieved in *Corynebacterium ammoniagenes* strains obtained after random mutagenesis experiments, which were capable of accumulating 7.5 g/L inosinic acid (Tomita et al., 1991). Production of inosine, which can be obtained from inosinic acid by one dephosporylation step, was demonstrated in *B. subtilis* (Asahara et al., 2010). The bacterium was engineered by initially deleting the genes coding for enzymes involved in the conversion of inosinic acid to GMP and AMP. Subsequently, expression of purine biosynthetic genes organized in the 12-gene *pur* cluster was deregulated by deletion of the gene encoding the regulator PurR and by removing a riboswitch. The resulting mutant strain was shown to produce 6 g/L inosine from 30 g/L glucose (Asahara et al., 2010). Enzymes suitable for the required conversion of inosine into inosinic acid were characterized *in vitro* (Liu et al., 2012) and were more recently also applied in a whole-cell biotransformation (Yuan et al., 2016). As an alternative strategy, inosinic acid can also be produced by deamination of AMP (Li et al., 2017).

### Oligopeptides and proteins

The E numbers catalog also includes oligopeptides and proteins, which serve as preservatives, sweeteners or antibiotics. Nisin (E234), an antibiotic oligopeptide consisting of 34 amino acids, is naturally produced by *L. lactis* and shows activity against Gram-positive bacteria (Severina et al., 1998). Nisin is encoded by the gene *nisA* and thus produced by ribosomes and not by nonribosomal peptide synthetases as many other peptide antibiotics (Kaletta and Entian, 1989). Strains of the natural producer *L. lactis* produced nisin by fermentation of milk or whey. It turned out that utilization non-filtrated milk whey allows for much higher nisin titers compared to filtrated milk whey. With different *L. lactis* strains nisin titers of 11.1 g/L were obtained (de Arauz et al., 2008). Metabolic engineering efforts toward increased production of nisin in *L. lactis* have been reviewed very recently (Özel et al., 2018).

Thaumatin (E957) is a mixture of six proteins (thaumatin I, II, III, a, b, and c, all consisting of 207 amino acids). These sweet-tasting proteins are produced in berries of the plant katamfe (*Thaumatococcus daniellii*) (Wel and Loeve, 1972), which grows in western and central Africa (Adansi and Holloway, 1975). Already in 1982, one of the thaumatin genes was functionally expressed in *E. coli* (Edens et al., 1982). Later, thaumatin II was produced in *Aspergillus awamori* strains as well as in *B. subtilis* by expression of a codon-optimized thaumatin II gene and subsequent protein secretion (Illingworth et al., 1988; Moralejo et al., 1999). Thaumatin production in *A. awamori* was improved by deleting a gene encoding the protease aspergillopepsin B and by simultaneous overexpression of *bipA* encoding an endoplasmic reticulum chaperone. With this strategy, a titer of 13 mg/L properly folded thaumatin II was obtained (Moralejo et al., 2002; Lombraña et al., 2004).

Due to its antimicrobial activity lysozyme (E1105) is used as a preservative in foodstuff. Lysozyme cleaves 1,4-β-bonds in peptidoglycan, which disturbs the integrity of the cell wall and leads to lysis of bacterial cells (Mir, 1977). Currently, commercially available lysozyme is obtained from egg white, which has several drawbacks, e.g., it requires laborious purification and can cause immunological problems in humans. As an alternative to extraction of lysozyme from egg white, recombinant *A. niger* was shown to be capable of producing this enzyme with a titer of 0.21 g/L (Gheshlaghi et al., 2005). In the recent years, there was increased interest in establishing microbial production of human lysozyme with higher activity compared to hen egg lysozyme, e.g., using *Pichia pastoris, E. coli, S. cerevisiae*, and *Kluyveromyces lactis* (Ercan and Demirci, 2016). Titers in the range of 0.03–0.13 g/L human lysozyme were obtained by expressing the human gene in microbial host organisms.

### Macrolide antimicrobials

**Figure 8:**
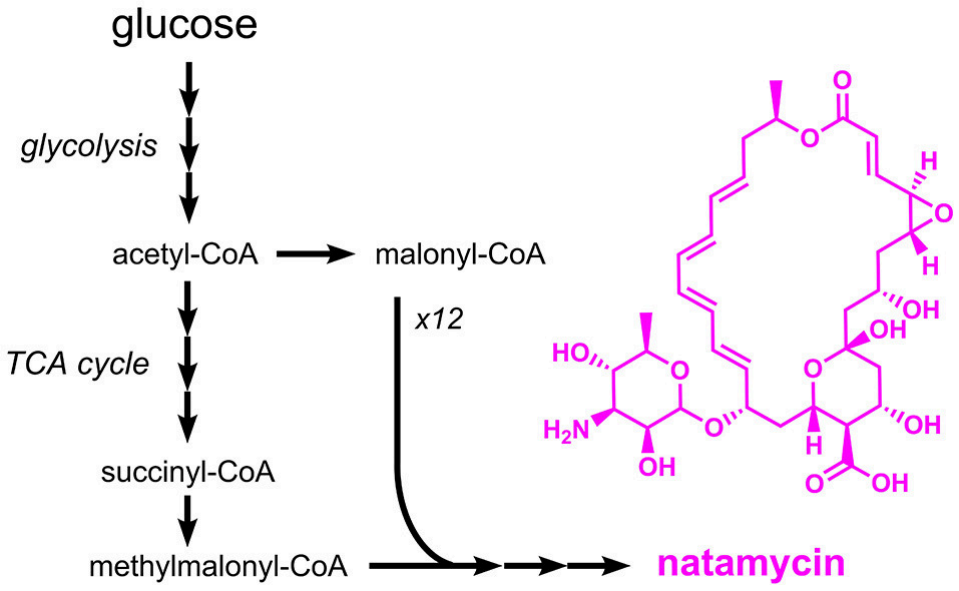
Overview on the biosynthetic pathway for the macrolide antibiotic natamycin. Three arrows indicate several reaction steps which are not shown in detail.

Natamycin (E235), a macrolide antibiotic from *Streptomyces natalensis*, has a similar mechanism of action as nisin (formation of pores in cell membranes), but acts exclusively against fungi, e.g. *Candida, Aspergillus* and *Penicillium* species (Pedersen, 1992). Natamycin is approved as a preservative for the surface treatment of hard or semi-hard cheese. It is also used for dried and salted sausages. Although nisin and natamycin share a similar mechanism of action, both compounds are structurally and metabolically unrelated. Production of natamycin is initiated by a polyketide synthase which uses methylmalonyl-CoA and 12 molecules of malonyl-CoA as substrates (Liu et al., 2015) (Figure 8). The resulting compound, referred to as natamycinolide, is further modified by carboxylation, glycosylation, and epoxidation, which give rise to natamycin. The biosynthesis pathway for natamycin is highly complex and (amongst others) involves the large polyketide synthases PimS1 and PimS2 with a length of 6,797 and 9,507 amino acids, respectively. Thus, it is not surprising that production of natamycin focused on natural producers such as *S. natalensis* and closely related species. The natamycin production capabilities of wild-type *S. natalensis* were optimized by testing different sources of carbon, nitrogen and phosphate. With glucose, potassium dihydrogen phosphate and a mixture of beef extract and yeast extract production of 1.5 g/L natamycin was observed (Farid et al., 2000). In a related study, it was tested whether the supplementation of short-chain acids or alcohols has a positive impact on natamycin production. As a result, it turned out that 1-propanol supplementation increased the natamycin titer to 10.4 g/L, which was 17% higher than the control strain without supplementation (Li et al., 2014a). The same effect was also observed for the supplementation of acetic acid and propionic acid (Elsayed et al., 2013). In this study a final titer of 4.0 g/L natamycin was achieved. Natamycin production in the natural producer *Streptomyces gilvosporeus* was improved by coupling the expression of the natamycin biosynthetic gene cluster to the expression of a kanamycin resistance gene (Wang et al., 2016). After seven iterative rounds of mutagenesis and selection for increased kanamycin resistance a strain producing 14.4 g/L natamycin was obtained.

## Discussion

Forty-two of the 316 compounds currently approved as food additives by the EU can in principle be produced in microorganisms until today (Table 1). It is impressive that a broad range of different compounds ranging from small organic acids to more complex secondary metabolites or polymers such as oligopeptides can now be accessed by tailor-made microbial cell factories (Table 2). Engineering efforts toward metabolite overproduction follow some basic strategies or principles, which generally apply for all tested microorganisms. These include increasing the activity of enzymes catalyzing rate-limiting reactions, the abolishment of natural regulatory circuits, e.g., transcriptional control of gene expression or allosteric feedback inhibition mechanisms, and the elimination of complex networks of competing pathways for preventing side product formation. Strain modification also targets increased carbon source uptake or product export or an increased resistance of the host against the product or a pathway intermediate. In addition to increasing product titers there is often also room for reduction of production costs. This can e.g., be achieved by stable integration of heterologous genes into the host genome and expression from constitutive promoters, which allows for avoiding the use of antibiotics (for plasmid maintenance) and inducer compounds. In several cases, changing the expression rate of endogenous genes can be superior to gene deletion. This is especially true when deletions lead to auxotrophic strains, which can be circumvented by downregulating the gene instead of deleting it.

**Table 2:** Production of food additives approved by the EU in engineered microorganisms.

**E number**	**Compound**	**Production host**	**Carbon source or precursor**	**Product titer (g/L)**	**Yield (g/g)**	**References**
**CARBOXYLIC ACIDS AND SHORT-CHAIN ALCOHOLS**						
E270	Lactic acid	*Bacillus* sp.	Glucose	225	0.99	Meng et al., 2012
E296	Malic acid	*Aspergillus oryzae*	Glucose	154	1.02	Brown et al., 2013
E297	Fumaric acid	*Rhozipus oryzae*	Glucose	85	0.85	Cao et al., 1996
E330	Citric acid	*Aspergillus niger*	Glucose	157	0.98	Yin et al., 2017
E334	Tartaric acid	*Gluconobacter oxydans*	Sorbitol	2.1	0.11	Chandrashekar et al., 1999
E355	Adipic acid	*Escherichia coli*	Glycerol	2.5	0.12	Cheong et al., 2016
E363	Succinic acid	*Escherichia coli*	Glycerol	96	0.96	Grabar et al., 2014
E422	Glycerol	*Candida glycerinogenes*	Glucose	130	0.63	Zhuge and Fang, 1995
E1520	1,2-Propanediol	*Closrtidium thermosaccharolyticum*	Glucose	7.9	0.27	Cameron and Cooney, 1986
**AMINO ACIDS**						
E620	l-glutamic acid	*Corynebacterium glutamicum*	Glucose	100	0.6	Ault, 2004
E920	l-cysteine	*Escherichia coli*	Glucose	1.7	0.06	Takagi et al., 1999
**TERPENOIDS**						
E160a	β-Carotene	*Escherichia coli*	Glycerol	0.47	0.02	Yoon et al., 2009
E160d	Lycopene	*Escherichia coli*	Glycerol	1.4	0.04	Zhu et al., 2015
E161b	Lutein	*Muriellopsis* sp.	CO_2_	0.035	–	Del Campo et al., 2000
E161g	Canthaxanthin	*Dietzia natronolimnaea*	Glucose	0.008	3.2^*^10^−4^	Gharibzahedi et al., 2012
E307	α-Tocopherol	*Euglena gracilis*	Tyrosine, homogentisate	0.14	–	Tani and Tsumura, 1989
**ONE-RING AROMATICS AND POLYPHENOLS**						
E100	Curcumin	*Escherichia coli*	Ferulic acid (in rice bran pitch)	0.06	–	Katsuyama et al., 2008
E162	Betanin	*Saccharomyces cerevisiae*	Dextrose	0.017	8.5^*^10^−4^	Grewal et al., 2018
E163	Callistephin (an anthocyanin)	*Escherichia coli*	Glucose	0.010	5.0^*^10^−4^	Jones et al., 2017
E214	4-Hydroxybenzoic acid	*Corynebacterium glutamicum*	Glucose	36.6	0.31	Kitade et al., 2018
E310	Gallic acid	*Escherichia coli*	Glucose	20	0.12	Kambourakis et al., 2000
E1519	Benzyl alcohol	*Escherichia coli*	Glucose	0.14	0.008	Pugh et al., 2015
**SUGAR-DERIVED COMPOUNDS**						
E300	2-Keto-l-gulonic acid (ascorbic acid precursor)	*Gluconobacter oxydans*	Sorbitol	130	0.87	Saito et al., 1997
E420	Sorbitol	*Zymomonas mobilis*	Glucose + fructose	233	1.0	Bringer-Meyer and Sahm, 1991
E421	Mannitol	*Corynebacterium glutamicum*	Fructose	87	0.93	Bäumchen and Bringer-Meyer, 2007
E574	Gluconic acid	*Aspergillus niger*	Glucose	311	1.05	Lu et al., 2015
E967	Xylitol	*Kluyveromyces marxianus*	Xylose	312	0.99	Zhang et al., 2015a
E968	Erythritol	*Psuedozyma tsukubaensis*	Glucose	243	0.61	Jeya et al., 2009
**NUCLEOTIDE-DERIVED COMPOUNDS**						
E101	Riboflavin	*Bacillus subtilis*	Glucose	4.9	0.05	Wang et al., 2014
E630	Inosinic acid	*Corynebacterium ammoniagenes*	Glucose	7.5	0.15	Tomita et al., 1991
**OLIGOPEPTIDES AND PROTEINS**						
E234	Nisin	*Lactococcus lactis*	Glucose	11.1	–	de Arauz et al., 2008
E957	Thaumatin	*Aspergillus awamori*	Glucose	0.013	–	Moralejo et al., 2002
E1105	Lysozyme	*Aspergillus niger*	Glucose	0.21	–	Gheshlaghi et al., 2005
**ANTIBIOTICS**						
E235	Natamycin	*Streptomyces natalensis*	Complex ingredients	14.4	–	Wang et al., 2016

Interestingly, the range of host organisms exploited for production of food additives is not restricted to commonly used organisms such as *E. coli* and *S. cerevisiae*, but comprises a broad spectrum of bacteria, fungi, and microalgae (Table 2). Although it is reasonable to use established platform organisms, for which a wealth of metabolic engineering tools is available, it is also important to sample the natural diversity of microorganisms (Pei and Schmidt, 2018). This is in particular true for those organisms already naturally overproducing compounds of interest. Here, the development of tools for strain modification not only serves for increasing the overall product titers. It also contributes to extend our knowledge of the microbial physiology and its diversity and might enable identification of novel compounds or pathway intermediates. This information might also enable the use of alternative (or synthetic) pathways for production.

Currently, production approaches for approved food additives are at three different stages reflecting economical viability of large-scale production, which are designated stage I, II, and III here. The titers required for an economically viable production (corresponds to stage III) strongly depend on the complexity and value of the product, thus only a rough classification for each stage can be given here. For several products microbial production is already economically viable and industrial processes are well-established, e.g., for l-glutamate, riboflavin, l-ascorbic acid, succinic acid, and lactic acid, which can be classified as stage III. For these compounds product titers of 100 g/L or more are typically obtained and also methods and processes for product purification are established. Stage II comprises products, for which titers in the range of 10–100 g/L are obtained. For such products titers obtained with microorganisms are close to economical viability and it can be expected that industrial production is in reach. Examples for products at stage II are adipic acid and one-ring aromatics such as 4-hydroxybenzoic acid. As one example, the company Verdezyne opened a pilot plant for bio-based adipic acid production with yeast in 2011 (Tetzlaf, 2011). Current challenges during microbial overproduction of stage III and II compounds are metabolic imbalances in the producer strain (e.g., with regard to the availability of reducing equivalents or ATP), or a depletion of metabolites required for sufficient biomass formation, but also product toxicity and insufficent product export. These challenges can be addressed by additional strain engineering and by choosing suitable process conditions (e.g., using two stage cultivation strategies with a production phase decoupled from biomass formation).

Products with titers in the mg/L range belong for the most part to stage I. In particular for more complex plant-derived polyphenols and terpenoids titers obtained in engineered microorganism are often still too low for establishing production at larger scale and today most of these compounds are still obtained by extraction from plants containing the desired compounds in larger amounts. Major challenges during production of plant natural products with microorganisms are often directly associated to their bioactivity as many of them have anti-microbial activities or tend to react with oxygen or radicals (anti-oxidants). For these products the optimization of cultivation conditions should focus on preventing oxidation and on reducing toxicity for the production host.

As only naturally-occurring metabolic pathways were exploited for food additive production in engineered microorganisms all compounds discussed in the main section of this review article are natural compounds. This is somehow self-evident when taking into consideration that synthetic compounds can typically not be produced using natural metabolic pathways. On the contrary, this does not mean that only pathways evolved by nature must be followed for production. One recent example is the plant-derived stilbene resveratrol, which is naturally produced from the phenylpropanoid *p*-coumaric acid. For circumventing a bottleneck reaction at the stage of aromatic amino acids the functional reversal of a bacterial catabolic pathway was exploited as a novel route toward resveratrol production (Kallscheuer et al., 2016). In another study, the relaxed substrate specificity of enzymes was exploited for producing small organic compounds, which cannot be obtained by pathways evolved by nature (Cheong et al., 2016).

In the recent years there was an increasing interest in replacing synthetic food additives by their natural counterparts. This is due to that fact that many synthetic food additives were shown to have negative effects on human health. Scientists have been pointing out for years that synthetic dyes may be involved in the development of attention deficit hyperactivity syndrome (ADHD) (Stevens et al., 2013). Since July 2010, the EU has made the warning label “May affect the activity and attention of children” mandatory for all manufacturers using the controversial substances. The new regulation applies to the dyes tartrazine (E102), quinoline yellow (E104), yellow-orange S (E110), azorubine (E122), cochineal red (E124), and allurred (E129). Also negative effects on health of artificial sweeteners are currently discussed. Cyclamic acid is banned in the US and UK due its potential links to cancer, but it is still approved as food additive in the EU as E952. Also the role of artificial sweeteners in the prevention of obesity is controversially discussed (Suez et al., 2014).

In contrast, natural products approved by the EU are generally recognized as safe, well-accepted by customers and some even show health-promoting effects (Vauzour et al., 2010). It can be expected that future efforts for increasing microbial production of such natural compounds will profit from novel methods such as CRISPR/Cas9 and from combination of rational strain engineering, adaptive laboratory evolution and high-throughput screening approaches (Schallmey et al., 2014; Eggeling et al., 2015; Shalem et al., 2015). One promising example are transcription factor-based biosensors for screening of strain libraries, e.g., for production of l-lysine, succinic acid, adipic acid, and also for plant polyphenols such as naringenin or quercetin (Eggeling et al., 2015). It is highly likely that such novel synthetic biology approaches will render microbial production especially of many plant natural compounds economically viable within the next 10–15 years.

## Author contributions

The author confirms being the sole contributor of this work and approved it for publication.

### Conflict of interest statement

The author declares that the research was conducted in the absence of any commercial or financial relationships that could be construed as a potential conflict of interest.
